# Association between Genetic Variation in the Oxytocin Receptor Gene and Emotional Withdrawal, but not between Oxytocin Pathway Genes and Diagnosis in Psychotic Disorders

**DOI:** 10.3389/fnhum.2015.00009

**Published:** 2015-01-23

**Authors:** Marit Haram, Martin Tesli, Francesco Bettella, Srdjan Djurovic, Ole Andreas Andreassen, Ingrid Melle

**Affiliations:** ^1^NORMENT, KG Jebsen Centre for Psychosis Research, Institute of Clinical Medicine, University of Oslo, Oslo, Norway; ^2^Division of Mental Health and Addiction, Oslo University Hospital, Oslo, Norway; ^3^Department of Medical Genetics, Oslo University Hospital Ullevål, Oslo, Norway

**Keywords:** oxytocin, vasopressin, schizophrenia, psychotic disorders, single nucleotide polymorphisms, polygenic risk score

## Abstract

Social dysfunction is common in patients with psychotic disorders. Oxytocin is a neuropeptide with a central role in social behavior. This study aims to explore the relationship between oxytocin pathway genes and symptoms related to social dysfunction in patients with psychotic disorders. We performed association analyses between four oxytocin pathway genes (OXT, OXTR, AVP, and CD38) and four areas of social behavior-related psychopathology as measured by Positive and Negative Syndrome Scale. For this purpose, we used both a polygenic risk score (PGRS) and single OXTR candidate single nucleotide polymorphism previously reported in the literature (rs53576, rs237902, and rs2254298). A total of 734 subjects with DSM-IV psychotic spectrum disorders and 420 healthy controls were included. Oxytocin pathway PGRSs were calculated based on the independent Psychiatric Genomics Consortium study sample. There was a significant association between symptom of Emotional Withdrawal and the previously reported OXTR risk allele A in rs53576. No significant associations between oxytocin pathway gene variants and a diagnosis of psychotic disorder were found. Our findings indicate that while oxytocin pathway genes do not appear to contribute to the susceptibility to psychotic disorders, variations in the OXTR gene might play a role in the development of impaired social behavior.

## Introduction

Oxytocin and vasopressin are closely related neuropeptide hormones, with similarities in structure, gene locations, pharmacological properties (Manning et al., 2008), and with roles in relation to social behavior (Insel and Young, 2001); including social memory, pair bonding, and parental behavior (Ferguson et al., 2001; Jin et al., 2007). Administration of intranasal oxytocin in humans has indicated increased interpersonal trust (Kosfeld et al., 2005), lower anxiety levels and improved social cognition (Macdonald and Macdonald, 2010). An increasing number of studies have investigated the relationship between oxytocin- and vasopressin-related genes and social behavior, including trust-related clinical phenotypes. This includes the genes coding for the production of oxytocin (OXT), vasopressin (AVP), the oxytocin receptor (OXTR) in addition to CD38, a transmembrane protein important for oxytocin secretion in the central nervous system (Bartz and McInnes, 2007; Jin et al., 2007; Lopatina et al., 2012).

The OXTR polymorphisms rs53576 and rs2254298 are seen as particularly promising candidates (Meyer-Lindenberg et al., 2011; Kumsta and Heinrichs, 2013). Multiple studies have indicated the A allele of rs53576 as a potential risk allele for social impairment. This allele has been associated with increased risk for autism in a Chinese Han population (Wu et al., 2005), less maternal sensitivity (Bakermans-Kranenburg and van Ijzendoorn, 2008), lower dispositional and behavioral empathy (Rodrigues et al., 2009) and lower sociability, reliance on social approval and learning from interpersonal feedback (Tost et al., 2010), lower positive affect in male, healthy subjects (Lucht et al., 2009), more feelings of loneliness in healthy females (van Roekel et al., 2013), and lower trust behavior in healthy subjects (Krueger et al., 2013). Variants in rs2254298 are found to be associated with risk of autism spectrum disorders (ASD), amygdala volume, anxiety, and depression (Meyer-Lindenberg et al., 2011; Kumsta and Heinrichs, 2013). However, both a recent large scale study (N~11000) (Chang et al., 2014) and a recent meta-analysis (*N* = 48 studies and 17559 participants for rs53576 and 34 studies and 13547 participants for rs2254298) (Bakermans-Kranenburg and van Ijzendoorn, 2014) did not find any associations to trust-related behavior for these two single nucleotide polymorphism (SNP) when combining the original studies.

Psychotic disorders, in particular schizophrenia, are characterized by severe social dysfunction (Green and Phillips, 2004; Addington et al., 2006, 2010; Reichenberg, 2010; Schmidt et al., 2011; Kidd, 2013). The oxytocin–vasopressin system’s effects on social behaviors, and oxytocin’s potential as a treatment option in disorders with social dysfunctions (Viero et al., 2010; Pedersen et al., 2011; Mah et al., 2013), made oxytocin the focus of interest in regard to psychotic disorders (Fischer-Shofty et al., 2013b). Oxytocin plasma levels have been found to correlate with social cognitive measures in patients with schizophrenia and healthy controls (Keri et al., 2009; Walss-Bass et al., 2013; Rubin et al., 2014) as well as positive symptoms in schizophrenia (Rubin et al., 2014). Vasopressin plasma levels have been found to be reduced in both patients and relatives (Rubin et al., 2014) and associated with positive symptoms and cognitive impairments in subgroups of psychotic patients (Rubin et al., 2013). Oxytocin’s effect on social behavior has made it a particular candidate for the treatment of negative symptoms in schizophrenia (Davis et al., 2014; Millan et al., 2014).

A few case–control studies to investigate oxytocin gene variation as a potential risk factor for psychotic disorders have been undertaken, and some candidate risk SNPs have been suggested (Souza et al., 2010; Teltsh et al., 2011; Montag et al., 2012). However, these studies have been small and a recent meta-analysis was not able to detect any associations between OXTR SNPs and the risk of schizophrenia (Watanabe et al., 2012). OXTR SNPs have however been found to be associated with clinical symptomatology in patients with schizophrenia, including general psychopathology (rs53576 and rs2254298), negative symptoms as measured by the Positive and Negative Syndrome Scale (PANSS) (rs237902), and empathic concern (rs2254298) (Montag et al., 2012, 2013).

Based on the inconclusive results of the few previous molecular genetic studies in this area, our aims were to investigate the relationship between variations in oxytocin-related genes and social behavior-related psychopathology in a large patient sample with psychotic disorders [i.e., schizophrenia and bipolar disorders (BD)] using two different approaches. The first and most novel approach was to investigate a measure related to the overall effect of oxytocin pathway genes, a polygenic risk score (PGRS) for OXT, AVP, CD38, and OXTR polymorphisms. Since each single risk variant in psychotic disorders has a small effect on disease phenotypes, this is a method to estimate cumulative genetic risk. We here assign a PGRS to individual cases in an independent sample based on summary statistics from one discovery case–control sample. The PGRS can include a few genome-wide significant SNPs, millions of SNPs from the entire genome or SNPs with significance over a certain threshold in a specific pathway. The method can be used to predict both case–control status and to explore potential intermediate phenotypes (Purcell et al., 2009; Tesli et al., 2014).

We additionally wanted to replicate findings regarding SNPs found to be associated either to schizophrenia or social behavior-related psychopathology (OXTR SNPs rs53576, rs2254298, and rs237902) in several previous studies. In both cases, we investigated potential associations with symptom measures chosen *a priori* due to their basis in aspects of social behavior: i.e., suspiciousness/persecution (p6), hostility (p7), emotional withdrawal (n2), and passive/apathetic social withdrawal (n4) from the PANSS (Kay et al., 1987).

## Materials and Methods

### Ethics statement

The study was approved by the Regional Committee for Medical Research Ethics and the Norwegian Data Inspectorate and was conducted according to the principles of the Declaration of Helsinki. All participants were adequately informed of the background, purpose, methods, sources of funding, potential benefits, and discomforts as well as the further storage and use of the data collected in this study. Potential subjects were informed of their right to withdraw their consent at any time. Each subject provided their freely given, written informed consent prior to the collection of data. Some patients with schizophrenia or BD may have a reduced ability to give informed consent, but in the current study only participants with a capacity to consent were included. This was specifically assessed by the clinical recruitment teams, which included experienced clinical psychologists or psychiatrists, according to a procedure approved by the local Regional Committee for Medical Research Ethics. Potential participants who declined to participate were not disadvantaged in any way by not participating in the study, and received same quality and amount of treatment and care from the hospital as the participants.

### Sample characteristics

Participants were included in the Norwegian multicentre Thematically Organized Psychosis Research (TOP) study recruiting patients from in- and out-patient clinics in South-Eastern Norway. All participants in this part of the study were Caucasian and diagnosed with a psychotic spectrum disorder according to DSM-IV using Structured Clinical Interview for DSM-IV Axis 1 Disorders (SCID-1). None had a history of head injury, neurological disorder, autoimmune or infectious disorders or malignancies, or an IQ below 70. All patients were examined by a physician and fasting blood samples were drawn in the morning. Of the total sample of 734 with a schizophrenia PGRS, 731 also had genotyped or imputation data for the three OXTR SNPs of interest, and 725 had available symptom scores (Table 1). The sample of 734 patients covered the following diagnoses: schizophrenia (*n* = 265, out of which 262 had data for the SNPs of interest and 263 had symptom scores), schizophreniform disorder (*n* = 23), schizoaffective disorder (*n* = 62), BD I (*n* = 180), BD II (*n* = 72), BD not otherwise specified (*n* = 18), depressive psychosis (*n* = 21), and other psychosis (*n* = 93). Age- and sex-matched healthy controls were selected randomly from the same catchment area as the TOP sample and screened for the presence of severe mental disorders in the participant and in first-degree relatives. The control sample consisted of 420 subjects with genotyped or imputation data for SNPs of interest, out of which 417 had PGRSs. None had a history of head injury or neurological disorders, none were illicit drug users and finally none had medical problems that in some way could interfere with brain function.

**Table 1:** **Demographic and clinical background of the study sample included in PANSS analysis and healthy controls**.

Parameters	Patients		Controls	
	*N*	Mean ± SD	*N*	Mean ± SD
Age (years)	725	33.4 ± 11.2	411	34.7 ± 9.9
Gender (m/f)	725	361/364	412	208/204
Schizophrenia (N/%)	263/36.3%	–	–	
Education (mean years)	707	13.5 ± 2.9	–	
PANSS score p6	724	2.33 ± 1.5	–	
PANSS score p7	724	1.22 ± 0.6	–	
PANSS score n2	724	2.04 ± 1.2	–	
PANSS score n4	724	2.19 ± 1.4	–	

*PANSS, positive and negative syndrome scale*.

### Assessments

For assessment of social dysfunction, we used the PANSS (Kay et al., 1987). This scale measures psychopathology that is common in psychotic disorders for the last week: positive, negative, depressive, disorganized, and excitative symptoms by a 30-item, 7-point rating scale (Langeveld et al., 2013). The scale includes a thorough definition of the content and behavioral manifestations of each symptom/item with descriptions of each anchor point of the scale. The ratings are based on information elicited through a semi-structured interview and is based on the patients’ thought content as conveyed during the interview information from informants and clinical observation during the interview. For the current study, we pre-selected the four items that were clearly related to trust and social function as defined by their definitions; two positive symptoms (p) and two negative symptoms (n): Suspiciousness/Persecution (p6); e.g., unrealistic or exaggerated ideas of persecution, as reflected in guardedness, a distrustful attitude, suspicious hypervigilance, or frank delusions that others mean harm, Hostility (p7); e.g., verbal and non-verbal expressions of anger and resentment, including sarcasm, passive-aggressive behavior, verbal abuse, and assaultiveness, Emotional Withdrawal (n2); e.g., lack of interest in, involvement with, and affective commitment to life’s events and Passive/Apathetic Social Withdrawal (n4); e.g., diminished interest and initiative in social interactions due to passivity, apathy, anergy, or avolition that leads to reduced interpersonal involvements and neglect of activities of daily living.

### Gene selection and genotyping

As outlined above, we used two different approaches for the selection of genetic variants to answer our research questions. The first was to investigate an overall effect of oxytocin pathway genes by calculating a PGRS for schizophrenia for OXT, AVP, CD38, and OXTR polymorphisms using the results from an independent schizophrenia case–control study (Psychiatric Genomics Consortium (PGC), 2011). The second approach was to select SNPs previously associated to schizophrenia or to social behavior-related symptoms. For this purpose, the three OXTR SNPs, rs53576, rs2254298, and rs237902, were included. The sample was genotyped using the Affymetrix Genome-Wide Human SNP Array 6.0 (Affymetrix Inc., Santa Clara, CA, USA). Samples were excluded through quality control if they had low-yield (call rate below 97%), if they were duplicates of other samples, if they had a sex determined by X chromosome marker homozygosity different from their reported sex or if they were calculated to have other ancestry than European (Athanasiu et al., 2010). Markers with severe deviation from Hardy–Weinberg equilibrium, low-yield (<95%) or with minor allele frequencies below 0.05 were excluded.

### Imputation of SNPs

Following the above mentioned quality control, the candidate SNPs were imputed with MaCH [43]^1^ using the European samples in the Phase I release of the 1000 Genomes Project [SNPs not present in the 1000 Genomes reference, and SNPs with ambiguous strand alignments (A/T and G/C SNPs), were removed from the sample data sets]. Imputation was a three stage process, involving (I) ChunkChromosome where the data set was broken into 2500 SNP pieces with 500 SNP overlap^2^, (II) MaCH where each piece was phased (40 rounds and 400 states)^3^, and (III) Minimac where each phased piece was imputed to the 1000 Genomes European reference panel (20 rounds and 400 states)^4^. In the third stage, all imputed SNPs were provided with an estimated *r*^2^ score as quality metric. Exclusions were made of SNPs with an *r*^2^ score <0.5 and with a minor allele frequency <0.01 leaving 9,584,802 SNPs. The imputed SNPs used in the PGRS were pruned to obtain a set of independent alleles in linkage equilibrium at *r*^2^  < 0.25.

### Polygenic risk score

A PGRS for the schizophrenia phenotype was computed based on imputed SNPs following the method developed by Purcell and co-workers (Purcell et al., 2009) and described in details for the current sample elsewhere (Tesli et al., 2014). With the aid of PLINK version 1.07^5^ (40), we performed a meta-analysis including all substudies in the Psychiatric Genomics Consortium (PGC) (2011) *except* the current sample (TOP sample) (*n* = 9146 schizophrenia cases and 12111 controls), to obtain risk allele effect sizes [ln(OR)] for SNPs in the oxytocin pathway genes: OXT [OMIM 167050], OXTR [OMIM 167055], CD38 [OMIM 107270], and AVP [OMIM 192340]. The SNPs within each gene were subsequently pruned by grouping them in LD (*r*^2^  < 0.25) sets and selecting from each set the representative with the highest absolute effect size. This procedure left us with 32 SNPs: (rs11087565 and rs857240) in AVP; (rs10805347, rs17476066, rs1803404, rs3733593, rs3756243, rs3796878, and rs7655635) in CD38; (rs2326055, rs4815560, and rs6084253) in OXT; and (rs1008642, rs1042778, rs13087941, rs17049532, rs17049552, rs2072582, rs2268484, rs2268491, rs2268494, rs2268495, rs2270463, rs2270465, rs2301261, rs237860, rs237864, rs237888, rs237889, rs237899, rs6443206, and rs7629329) in OXTR. A cumulative risk score was then computed for each individual in the TOP sample summing up the effect sizes of the selected SNPs multiplied by the number of risk alleles carried by that individual. Such a cumulative risk score is assumed to represent an oxytocin pathway cumulative risk for schizophrenia.

### Statistical analysis

#### Polygenic risk score vs. psychotic phenotypes

Potential differences in mean schizophrenia oxytocin pathway PGRS between cases and healthy controls were first investigated with *t*-tests for independent samples (SPSS version 19). These analyses were performed for the entire psychosis spectrum as well as in schizophrenia cases only. The PGRSs were normally distributed according to visual inspection of histograms. Additionally, we performed linear regression analyses with the four PANSS scores [suspiciousness/persecution (p6), hostility (p7), emotional withdrawal (n2), and passive/apathetic social withdrawal (n4)] as dependent variables and with the PGRS as an independent variable (SPSS version 19) in the total patient sample, correcting for gender and diagnostic group (schizophrenia spectrum vs. affective spectrum). Because social dysfunction is particularly pronounced in schizophrenia, we did a follow-up analysis for the case–control sample including only patients meeting the diagnostic criteria for schizophrenia.

#### Single SNPs vs. psychotic phenotypes

The case–control association analysis between the SNP of interest (rs53576) and a psychotic disorder diagnosis was performed with an allele model and gender as covariate (PLINK version 1.07), with a follow-up analysis restricted to cases with schizophrenia vs. healthy controls. Statistical power was estimated with the Genetic Power Calculator^6^, assuming a disease prevalence of 0.01 for schizophrenia and 0.02 for psychotic disorders, an additive allelic model and a type I error rate of 0.05. The high risk allele frequency was 0.67 and the powers were estimated to be 0.51 for psychotic disorders and 0.34 for schizophrenia. We then undertook linear regression analyses with PANSS p6, p7, n2, n4 as dependent variables and the SNPs of interest [rs53576, rs2254298 (imputed SNPs), and rs237902 (genotyped SNP)] as independent variables (PLINK version 1.07), correcting for gender and diagnostic group. The polymorphisms were divided into three groups (homozygote minor allele, heterozygote, and homozygote major allele) and analyzed with an additive model.

Additionally, we ran multiple regression analyses for each PANSS item separately for the three candidate SNPs, to test for potential specificity of each SNP. These analyses were performed with the software R. Bonferroni correction was applied to both single SNP and multiple regression analyses.

## Results

### Polygenic risk score vs. psychotic phenotypes

There was no statistically significant association between the schizophrenia oxytocin pathway PGRS and either the diagnosis of psychotic disorders/schizophrenia or to psychopathology related to social behaviors as measured by the selected PANSS items. All associations between PGRS scores and clinical symptoms were low (coefficients ranging from 0.002 to 0.027/*p*-values ranging from 0.50 to 0.99).

### Single SNPs vs. psychotic phenotypes

In the study of SNPs of interest, we did not find any association between rs53576 and the risk of having a psychotic disorder *per se* or to schizophrenia in particular. We found a statistically significant association between rs53576 and the level of Emotional Withdrawal (n2) (nominal *p* = 0.007; *p* = 0.084 after Bonferroni correction taking into consideration the four PANSS items and the three SNPs). We did not find any associations between single SNPs and the other symptom scores (Table 2). The multiple regression analyses indicated a significant association between rs53576 and the level of Emotional Withdrawal (nominal *p* = 0.00225; *p* = 0.009 after Bonferroni correction taking into consideration the four PANSS items).

**Table 2:** **Association analysis between PANSS scores and oxytocin receptor polymorphisms**.

SNP	Nearest gene	Reference allele^a^	*N*	PANSS item	Beta	*P* (Bonferroni)	*P* multiple regression (Bonferroni)
rs53576	OXTR	G	725	P6	0.123	0.159 (1)	0.561 (1)
			724	P7	−0.032	0.387 (1)	0.230 (0.92)
			724	N2	−0.203	**0.007 (0.084)**	**0.00225 (0.009)**
			724	N4	−0.159	0.060 (0.72)	0.209 (0.836)
rs237902	OXTR	A	721	P6	0.145	0.055 (0.66)	0.160 (0.640)
			721	P7	−0.016	0.574 (1)	0.982 (1)
			721	N2	0.018	0.791 (1)	0.238 (0.952)
			721	N4	−0.047	0.529 (1)	0.822 (1)
rs2254298	OXTR	G	725	P6	0.008	0.941 (1)	0.973 (1)
			724	P7	0.055	0.909 (1)	0.739 (1)
			724	N2	0.054	0.573 (1)	0.992 (1)
			724	N4	0.015	0.890 (1)	0.507 (1)

*SNP, single nucleotide polymorphism; OXTR, oxytocin receptor gene; PANSS, positive and negative syndrome scale*.

*The multiple regression analyses include all three SNPs, sex and diagnosis as predictor variables for each PANSS item*.

*^a^An additive model is used, where the Beta value refers to the increase in the dependent variable for each added reference allele*. *Bold font means significant after Bonferroni correction*.

## Discussion

The main finding of the current study was a statistically significant association between rs53576 in OXTR, frequently found to be associated with social behavior in previous studies, and the level of Emotional Withdrawal, a negative symptom frequently occurring in psychotic disorders. More specifically; we found that having the risk allele A in rs53576 was associated with higher scores on Emotional Withdrawal as measured by the PANSS. Emotional Withdrawal is characterized by the lack of interest in, involvement with and affective commitment to life’s events and is seen in a wide range of psychiatric diseases. This finding thus supports previous ones of a negative association with social behavior for the A allele for areas covering maternal sensitivity (Bakermans-Kranenburg and van Ijzendoorn, 2008), empathy (Rodrigues et al., 2009), sociability (Tost et al., 2010), trust behavior in healthy subjects (Krueger et al., 2013), and seeking of emotional support in times of distress (Kim et al., 2010). Contrasting studies have however indicated that schizophrenia patients carrying the G allele had higher general psychopathology scores measured by PANSS (Montag et al., 2013) and ADHD children carrying the A allele had better social cognitive ability (Park et al., 2010). These could be in line with a “flip-flop phenomenon” (Lin et al., 2007) or influenced by different ethnic sample backgrounds (Propper et al., 2007). Or they could also be spurious findings due to small samples and low statistical power (Button et al., 2013) as indicated by the meta-analyses (Psychiatric Genomics Consortium (PGC), 2011; Bakermans-Kranenburg and van Ijzendoorn, 2014). We did not find any associations to other clinical symptoms. This could partly be due to a low mean value for the symptom measures in question.

Only a few case–control studies have investigated the relationship between the diagnosis of schizophrenia and oxytocin-related genes, using different strategies for SNP selection in the oxytocin pathway genes and the results appear inconclusive (Souza et al., 2010; Teltsh et al., 2011; Watanabe et al., 2012; Montag et al., 2013). We did not find any associations between oxytocin pathway genes, including in the form of PGRS for the oxytocin pathway, and the diagnosis of a psychotic disorder. This is in line with findings from the PGC schizophrenia sample where no SNPs in these four genes remained statistically significant after Bonferroni gene-wide correction (published at http://www.broadinstitute.org/mpg/ricopili/).

Due to overlapping symptomatology and susceptibility genes across severe mental disorders (Levy and Manove, 2012; Craddock and Sklar, 2013; Kidd, 2013) we here sought to include a wide range of diagnoses and due to evidence of shared clinical symptoms, genetic risk (Andreassen et al., 2013), neurocognitive impairments (Simonsen et al., 2011) as well as brain abnormalities (Rimol et al., 2010; Brandt et al., 2014) we also included patients with BD. This could potentially weaken associations. A follow-up analysis focusing only on patients with schizophrenia did not however change the results.

While the current sample is large compared to previous samples with structured information on clinical symptomatology, it is still on the smaller side compared to the consortia based case–control GWAS samples. We thus also run the risk of both type I and type II errors, which is why we have chosen to present both nominal and Bonferroni-corrected significance levels (Table 2), as a full Bonferroni correction is based on the assumption of complete independence between measures.

So to conclude: our findings suggest that oxytocin-related genes are not main contributors to the risk of developing schizophrenia or other psychotic disorders, but support the notion that specific variation in the OXTR gene might play a role in the magnitude of altered social behavior observed in patients with these disorders. Theoretically, genetic or environmental risk factors can be associated with the risk for a particular disease phenotype in addition to – or independent from – the risk for the disorder *per se* (Aas et al., 2012; Varese et al., 2012; Pranavchand and Reddy, 2013). The effect of medication is also often unrelated to the genetic underpinnings of the disorder, and could explain why intra-nasally administered oxytocin have a symptomatic effect in patients with schizophrenia (Bujanow, 1972; Feifel et al., 2010; Pedersen et al., 2011; Fischer-Shofty et al., 2013a; Lee et al., 2013; Modabbernia et al., 2013) even if schizophrenia appears to not be related to oxytocin genetic variants. In light of a proposed interactionist model suggesting that the treatment effect of oxytocin is dependent on personality traits and context (Bartz et al., 2011; Luminet et al., 2011; Cardoso et al., 2012; Strathearn et al., 2012), our finding suggests that the level of negative symptoms, in particular Emotional Withdrawal, should be of interest in further oxytocin trials in this patient group.

## Author Contributions

Marit Haram contributed to the conception, analysis and interpretation of data, and drafted and revised the work, finally approved the version to be published and agreed to be accountable for all aspects of the work. Martin Tesli contributed to the conception, analysis and interpretation of data, and drafted and revised the work, finally approved the version to be published and agreed to be accountable for all aspects of the work. Francesco Bettella contributed to the analysis and interpretation of data, drafted, and revised the work, finally approved the version to be published and agreed to be accountable for all aspects of the work. Srdjan Djurovic contributed to the acquisition and interpretation of data, revised the work, and finally approved the version to be published and agreed to be accountable for all aspects of the work. Ole Andreas Andreassen contributed to the conception, acquisition and interpretation of data, revised the work, and finally approved the version to be published and agreed to be accountable for all aspects of the work. Ingrid Melle contributed to the conception, acquisition and interpretation of data, drafted and revised the work, and finally approved the version to be published and agreed to be accountable for all aspects of the work.

## Conflict of Interest Statement

Ole Andreas Andreassen is involved in a project investigating oxytocin effects sponsored by OptiNose AS and partly funded by BIA grant from Research Council of Norway. The other authors declare no conflict of interest concerning this manuscript.

## Footnotes

^1^http://www.sph.umich.edu/csg/abecasis/MACH/download/1000G-PhaseI-Interim.html

^2^http://genome.sph.umich.edu/wiki/ChunkChromosome

^3^http://www.sph.umich.edu/csg/abecasis/MaCH/download/

^4^http://genome.sph.umich.edu/wiki/Minimac

^5^http://pngu.mgh.harvard.edu/purcell/plink/

^6^http://pngu.mgh.harvard.edu/~purcell/gpc/
